# The relationship between perfectionism, self-perception of orofacial appearance, and mental health in college students

**DOI:** 10.3389/fpubh.2023.1154413

**Published:** 2023-05-05

**Authors:** Xinlin Gao, Jiawei Zhong, Hanchao Li, Yapeng Pei, Xixin Li, Siyu Zhang, Yuan Yue, Xin Xiong

**Affiliations:** ^1^Department of Orthodontics, National Clinical Research Center for Oral Diseases, State Key Laboratory of Oral Diseases, West China Hospital of Stomatology, Sichuan University, Chengdu, China; ^2^Department of Prosthodontics, National Clinical Research Center for Oral Diseases, State Key Laboratory of Oral Diseases, West China Hospital of Stomatology, Sichuan University, Chengdu, China; ^3^Department of Temporomandibular Joint, National Clinical Research Center for Oral Diseases, State Key Laboratory of Oral Diseases, West China Hospital of Stomatology, Sichuan University, Chengdu, China

**Keywords:** perfectionism, psychological health, self-perception, orofacial appearance, body image

## Abstract

**Background:**

In dental clinical practice, self-perception of orofacial appearance is highly correlated with treatment satisfaction. Therefore, it is important to explore factors correlated with self-perception of orofacial appearance. Perfectionism may be one such factor. This study investigated the role of perfectionism in self-perception of orofacial appearance.

**Methods:**

Participants completed an online questionnaire that included demographic data, a measure of perfectionism, a measure of self-perception of orofacial appearance (including body image, smile appearance concern, and self-esteem), and a measure of anxiety and depression.

**Results:**

High perfectionism scores significantly predicted greater age, body image, smile appearance concern, and mental health scores and lower self-esteem scores (*p* < 0.005). After adjusting for possible confounding variables, smile appearance concern largely disappeared. Mental health acted as a mediator in the relationships between perfectionism and three orofacial appearance characteristics.

**Conclusion:**

High perfectionism predicted higher self-perception of body image, and lower mental health and self-esteem in college students. Mental health could mediate the relationships between perfectionism and self-perception of orofacial appearance.

## 1. Introduction

Perfectionism can be regarded as a personality trait exemplified by striving for perfection in everything, tending to set extremely high standards and being exceedingly sensitive to critical evaluation (1, 2). Numerous studies have found that perfectionism is correlated with eating disorders, oral health-related behaviors, will to get succeed, and self-perception of appearance (3–5). Excessively high perfectionism is associated with orthorexia nervosa, severe psychological problems, and even suicide (6, 7).

Esthetics refers to a set of principles governing the appreciation of beauty (8). Orofacial aesthetics is a type of aesthetics that is focused on one’s appearance, and has been found to correlate with some mental problems and various aspects of social life. In dental clinical work, patients with positive self-perception of orofacial appearance would show better adherence to the treatment plan and more satisfaction with the procedure and the effects of the therapy (8, 9). Conversely, low satisfaction with dental treatment outcomes may result in low ratings of trust between dentists and patients.

Faced with many challenges during the transition from school to society and from adolescence to adulthood, college students engage in quite intense mental activity and self-exploration, often experiencing mental illnesses (10). The influence of perfectionism on many aspects of young people’s self-evaluation such as in self-liking, self-competence, self-control, and self-confidence, is becoming clear (5, 11). However, it is still unclear how perfectionism correlates with college students’ self-perception of orofacial appearance and mental health.

This correlational study focused on exploring the relationship between perfectionism, self-perception of orofacial appearance (measured as body image, smile appearance concern, and self-esteem), and mental health (specifically anxiety, and depression). It was hypothesized that perfectionism would be highly correlated with greater self-perception of orofacial appearance and greater mental illness. Mental health would act as a mediating role in the relationships between perfectionism and self-perception of orofacial appearance. The results could help clinicians to assess patients’ psychological status when developing a treatment plan.

## 2. Methods

### 2.1. Study design

A cross-sectional study was designed to measure the correlations among the three variables. Ethic approval for the study was obtained from West China School of Stomatology Sichuan University Medical Ethics Committee (Code: WCHSIRB-CT-2022-241). The questionnaire was delivered to the college students through a link^1^ from May to June 2022. After reading the study information and agreeing to the informed consent page, they could then proceed to the questionnaire.

### 2.2. Research tools

The following research tools were used: the short-form Hewitt Multidimensional Perfectionism Scale-15 (12), the orofacial appearance perception questionnaire (8), the four-item patient health questionnaire-4 (13), and a demographic survey.

#### 2.2.1. The short-form Hewitt multidimensional perfectionism scale-15 (MPS-H15)

The MPS-H15 contains 15 items with a seven-point answer scale from strongly disagree (1) to strongly agree (7) (12). The Cronbach’s Alpha is 0.919. Higher scores indicate greater perfectionism. The scale measures three dimensions of perfectionism: self-oriented perfectionism (SOP), other-oriented perfectionism (OOP), and socially-prescribed perfectionism (SPP). Each dimension includes 5 questions.

SOP includes behaviors such as setting oneself unattainable goals and being stuck in stringent self-evaluation. OOP addresses having critical standards for other people and attaching great importance to others being perfect. SPP reflects the need to strive for perfection and to be perfect to others (14).

#### 2.2.2. The orofacial appearance perception questionnaire

The original OAP questionnaire consists of 17 items with a five-point Likert scale (1 = strongly disagree; 5 = strongly agree) measuring self-perception of orofacial appearance. The questionnaire measures four dimensions: body image, smile appearance concern, self-esteem, and perfectionism. However, the present study extracted the former three sections and replaced the brief perfectionism subscale with a more comprehensive questionnaire (MPS-15) to evaluate perfectionism more thoroughly. The Cronbach’s Alpha for the rest three subscales (13 items) is 0.797, 0.867, and 0.874, respectively.

Body image measures how much importance participants attached to their physical appearance including orofacial appearance. Smile appearance concern measures participants’ satisfaction (reverse coded) and affective factors (reverse coded) related to smile aesthetics (aesthetic anxiety, mental stress, and dental self-confidence). Self-esteem measures confidence in self-performance (reverse coded) (8).

#### 2.2.3. The 4-item patient health questionnaire-4

The PHQ-4 is an effective and convenient scale for measuring anxiety and depression, with items such as “Over the last 2 weeks, how often have you been bothered by the following problems?” Responses are scored as 0 (“not at all”), 1 (“several days”), 2 (“more than half the days”), or 3 (“nearly every day”) (15). Higher scores indicate greater mental illness.

#### 2.2.4. Sociodemographic variables

This study included variables such as age, sex, gender, *per capita* monthly household income, and major.

### 2.3. Participants

Participants who aged 17–28 and offered informed consent were included in the present study. Exclusion criteria: (1) cognitive impairment and illiteracy, (2) answers to all questions were illogical, and (3) spending insufficient time on the questionnaire.

### 2.4. Sample size calculation

Sample size was calculated using G*Power software (G*Power, Version 3.1.9.7) by prior power analysis. Student’s *t*-test between two independent means with a power of 95%, an alpha level = 0.05 (2-tailed), and a moderate effect size (*d* = 0.50) were entered, resulting in a minimum sample size needed of 210 participants (16).

### 2.5. Statistical analysis

R (http://www.R-project.org) and EmpowerStats software (www.empowerstats.com, X&Y solutions, China), and SPSS (version 25.0) were used for all analyses. *p* < 0.05 was regarded to be statistically significant. Means with standard deviations and percentages were used to describe continuous and categorical variables, respectively. A chi-square test was used to analyze the differences between groups of categorical variables. Univariate and stratified analysis and logistic regression analysis were performed to investigate the relationship between perfectionism and body image, smile appearance concern, self-esteem, and mental health before and after adjusting for other confounds. Mediation analysis was used to test the mediating role of mental health.

## 3. Results

### 3.1. Descriptive statistics

A total of 510 responses were collected for this study, 33 of which were excluded because they did not meet the age requirement, and 42 of which were also excluded because they took insufficient time to complete the questionnaire. The resulting effective rate was 85%. The final sample consisted of 435 college students from 17 to 28 years old (*M* = 20.4, SD = 2), most of whom were female (*n* = 303, 70%). Compared with male group, female participants had lower perfectionism scores (*p* < 0.001). Most participants had treatment experience regardless of male or female, the percentages were 63.6 and 74.2%, respectively. (See Table 1).

**Table 1:** Descriptive statistics of the characteristics of college students.

Gender	Male	Female	*p*-value
*N*	132	303	
Age	20.35 ± 1.91	20.35 ± 2.04	0.703
Perfectionism orientation			
SOP	22.85 ± 5.93	22.15 ± 5.86	0.152
OOP	21.35 ± 6.00	20.15 ± 5.49	<0.05
SPP	22.53 ± 5.67	21.01 ± 5.64	<0.01
Perfectionism scores	66.73 ± 15.92	66.30 ± 15.01	<0.05
Body image	16.32 ± 2.47	16.11 ± 2.42	0.349
Smile appearance concern	12.36 ± 3.94	12.55 ± 3.87	0.702
Self-esteem	16.45 ± 4.60	16.47 ± 4.32	0.979
PHQ-4	7.70 ± 3.14	8.15 ± 2.91	0.071
Dental treatment ever			<0.05
YES	84 (63.64%)	225 (74.26%)	
NO	48 (36.36%)	78 (25.74%)	
*Per capita* monthly household income, yuan			0.757
<3,000	35 (26.52%)	86 (28.38%)	
≥3,000	97 (73.48%)	217 (71.62%)	
Major			0.313
Medical	47 (35.61%)	93 (30.69%)	
Not medical	85 (64.39%)	210 (69.31%)	

Results in the table: Mean + SD/*N*(%). PHQ-4, the patient health questionnaire-4; SOP, self-oriented perfectionism; OOP, other-oriented perfectionism; SPP, socially-prescribed perfectionism.

### 3.2. Univariate analysis

Age and body image, smile appearance concern, self-esteem and PHQ-4 scores were significantly correlated with perfectionism scores (*p* ≤ 0.001) (see Table 2). Participants with one more perfectionism score had 0.05 higher body image scores (*β* = 0.05, *p* < 0.0001), 0.04 higher smile appearance concern scores (*β* = 0.04, *p* < 0.0001), and 0.04 higher PHQ-4 scores (*β* = 0.04, *p* < 0.0001). In contrast, they had 0.09 lower self-esteem scores (*β* = −0.09, *p* < 0.0001).

**Table 2:** Crude association between the perfectionism scores and other characteristics.

Characteristics	Perfectionism scores	
	*β*	*p*-value
Age	0.03 (0.01, 0.04)	<0.001
Body image	0.05 (0.03, 0.06)	<0.001
Smile appearance concern	0.06 (0.04, 0.09)	<0.001
Self-esteem	−0.09 (−0.12, −0.07)	<0.001
PHQ-4	0.04 (0.02, 0.06)	<0.001

Results in the table: *β* (95%CI). CI, confidence interval; PHQ-4, the patient health questionnaire-4.

### 3.3. Stratification analysis

**Figure 1:**
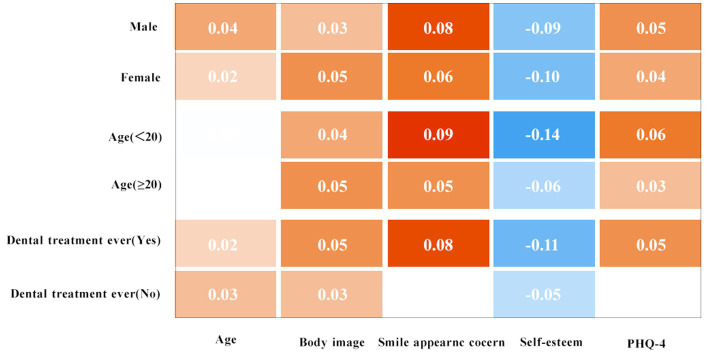
Cartesian thermal diagram showing *β* in stratification analysis. Orange means *β* > 0 and *p* < 0.05 between different perfectionism groups in the stratification. The deeper the orange is, the larger *β* is; blue means that *β* < 0 and *p* < 0.05 between different perfectionism groups in the stratification. The deeper the blue is, the larger the absolute value of *β* is; space means no significant differences exist between different perfectionism groups in the stratification.

The patients were stratified by sex (male or female), age (<20 or ≥20) and dental treatment ever (yes or no) groups. After stratifying by sex and age, the study found that in all the groups, the associations between perfectionism scores and other characteristics were significant. (See Table 3A; Figure 1).

**Table 3A:** Stratification analysis for associations between the perfectionism scores and other characteristics.

Characteristics	Gender		Age	
	Male	Female	<20	≥20
*N*	132	303	162	273
Age	0.04 (0.02, 0.06)***	0.02 (0.01, 0.04)***		
Body image	0.03 (0.01, 0.06)*	0.05 (0.03, 0.07)**	0.04 (0.02, 0.06)***	0.05 (0.03, 0.07)***
Smile appearance concern	0.08 (0.04, 0.12)***	0.06 (0.03, 0.09)***	0.09 (0.06, 0.12)***	0.05 (0.02, 0.08)**
Self-esteem	−0.09 (−0.14, −0.05)***	−0.10 (−0.13, −0.07)***	−0.14 (−0.18, −0.10)***	−0.06 (−0.10, −0.03)***
PHQ-4	0.05 (0.01, 0.08)**	0.04 (0.02, 0.06)***	0.06 (0.03, 0.08)***	0.03 (0.01, 0.06)*

Stratified by whether participants had ever received dental treatment, significant associations were found between perfectionism scores and other characteristics in the dental treatment group, but were not found in smile appearance concern and PHQ-4 stratifications in the non-dental treatment group (see Table 3B; Figure 1).

**Table 3B:** Stratification analysis for associations between the perfectionism scores and other characteristics.

Characteristics	Dental treatment ever	
	Yes	No
*N*	309	126
Age	0.02 (0.01, 0.04)**	0.03 (0.01, 0.05)***
Body image	0.05 (0.03, 0.07)***	0.03 (0.01, 0.06)*
Smile appearance concern	0.08 (0.05, 0.11)***	0.02 (−0.02, 0.07) 0.3014
Self-esteem	−0.11 (−0.14, −0.08)***	−0.05 (−0.11, −0.00)*
PHQ-4	0.05 (0.03, 0.07)***	0.02 (−0.02, 0.05) 0.3805

Results in the table: *β* (95%CI). PHQ-4, the patient health questionnaire-4. *p < 0.05, **p < 0.01, ***p < 0.001.

### 3.4. Logistic regression analysis

**Figure 2:**
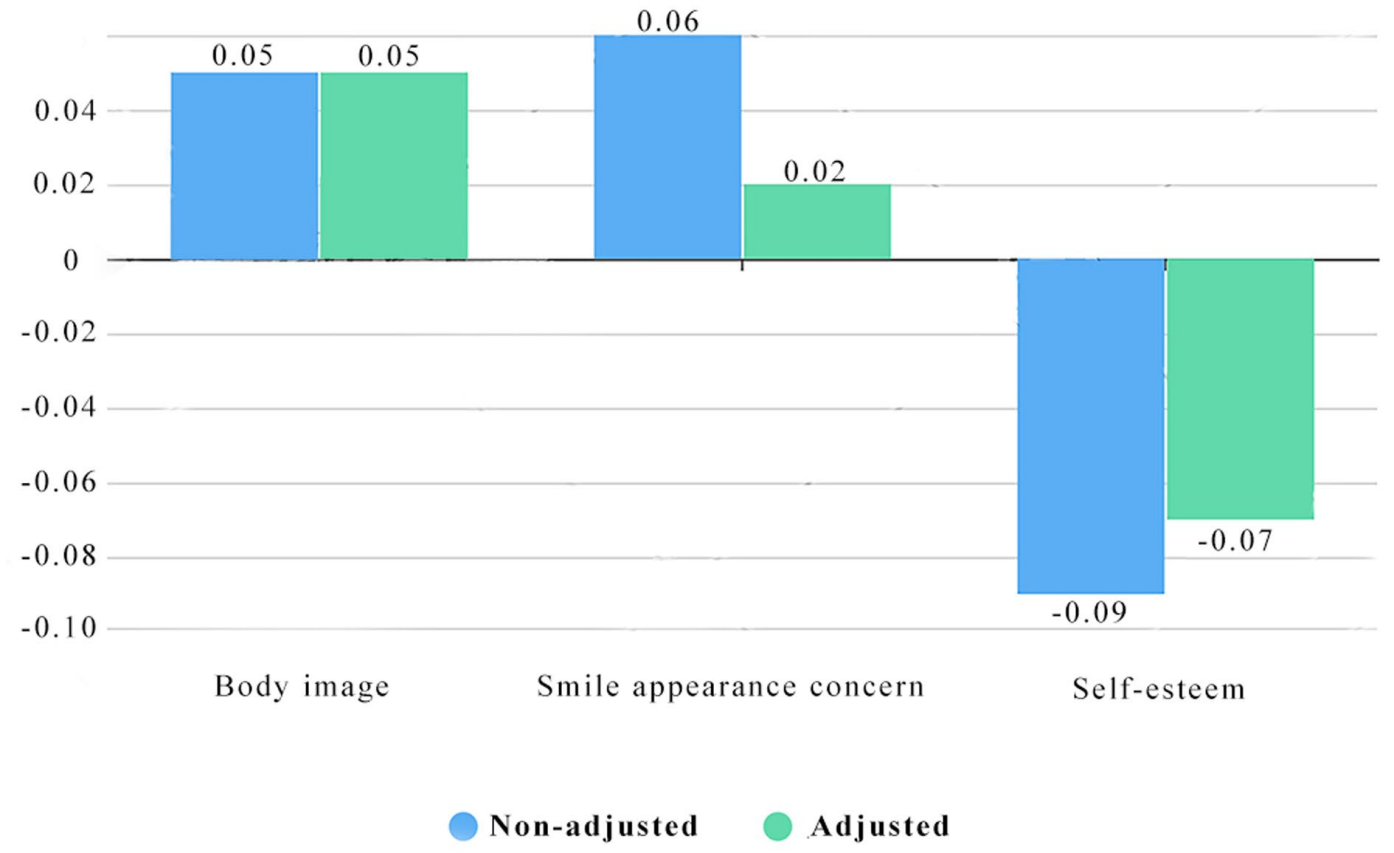
Logistic regression for associations between the perfectionism scores and other characteristics in college students.

After adjusting for confounds, the *β*s of perfectionism on body image and self-esteem were still statistically significant, suggesting the two characteristics are stable and reliable. The effect size of smile appearance concern changed from 0.06 to 0.02, resulting in the statistical significance disappearing after the adjustment (see Table 4; Figure 2).

**Table 4:** Logistic regression for the associations between perfectionism scores and other characteristics in college students.

Characteristics	Perfectionism scores
		*β*	95%CI	*p*-value
Body image	Non-adjusted		0.05	0.03, 0.06	<0.0001
	Adjusted^a^		0.05	0.03, 0.07	<0.0001
Smile appearance concern	Non-adjusted		0.06	0.04, 0.09	<0.0001
	Adjusted^b^		0.02	−0.01, 0.04	0.1537
Self-esteem	Non-adjusted		−0.09	−0.12, −0.07	<0.0001
	Adjusted^c^		−0.07	−0.09, −0.05	<0.0001

a Adjusted for age, sex, per capita monthly household, dental treatment ever, smile appearance concern, and self-esteem.

b Adjusted for age, sex, per capita monthly household, dental treatment ever, body image, and self-esteem.

c Adjusted for age, sex, per capita monthly household, dental treatment ever, body image, and smile appearance concern.

### 3.5. Pathway analysis

**Figure 3:**
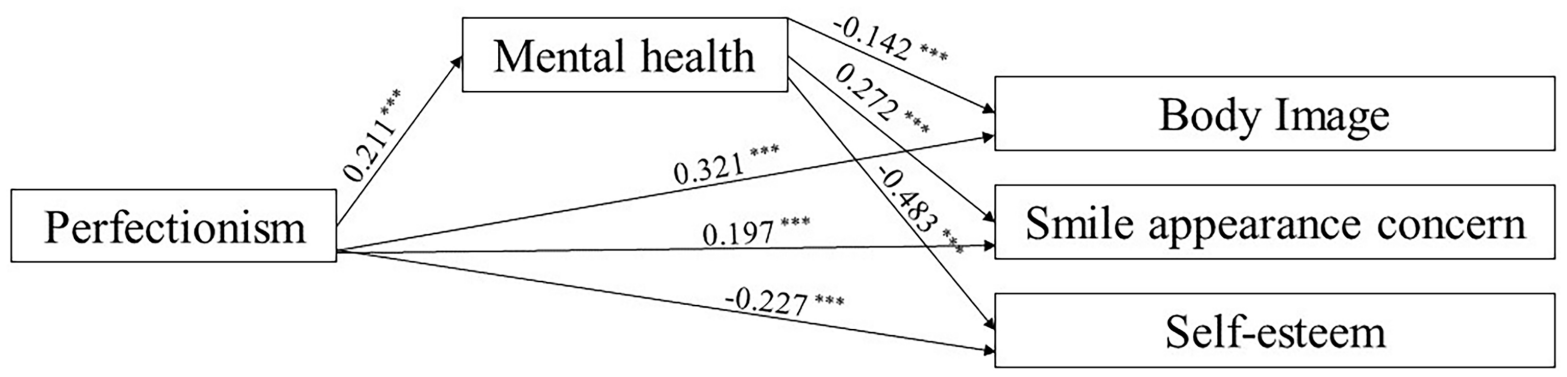
Structural model of median analysis.

The total and direct effects of perfectionism and mental health on body image, smile appearance concern and self-esteem were statistically significant. The indirect effects of mental health on the relationships between perfectionism and the other three characteristics were also of statistical significance. The effect of mental health explained 10.30, 22.60, and 31.07% of the relationships between perfectionism and body image, smile appearance concern and self-esteem, respectively (See Table 5; Figure 3).

**Table 5:** Median analysis results for the relationship between perfectionism and other characteristics.

Routine of pathway analysis	Effect size	*t*	Percentage
Perfectionism on body image via mental health			
Total effect	0.291	6.320***	
Indirect effect	−0.030		10.30%
Direct effect			
Perfectionism → body image	0.321	6.879***	
Perfectionism → mental health	0.211	4.488***	
Mental health → body image	−0.142	−3.046**	
Perfectionism on smile appearance concern via mental health			
Total effect	0.254	5.467***	
Indirect effect	0.057		22.60%
Direct effect			
Perfectionism → smile appearance concern	0.197	4.300***	
Perfectionism → mental health	0.211	4.488***	
Mental health → smile appearance concern	0.272	5.933**	
Perfectionism on self-esteem via mental health			
Total effect	−0.328	−7.236***	
Indirect effect	−0.102		31.07%
Direct effect			
Perfectionism → self-esteem	−0.227	−5.628***	
Perfectionism → mental health	0.211	4.488***	
Mental health → self-esteem	−0.483	−12.000**	

## 4. Discussion

The hypothesis that perfectionism would be highly correlated with greater self-perception of orofacial appearance and greater mental illness, and mental health would play a partial mediating role in the relationships between perfectionism and self-perception of orofacial appearance was supported. Higher perfectionism could predict higher body image, higher smile appearance concern, and lower self-esteem. Mental health explained 10.30, 22.60, and 31.07% of the relationships, respectively.

The study found that people with high perfectionism tended to be older than those with low perfectionism, which was consistent with previous research results (17, 18). Presumably, students in higher grades are under more academic pressure, and excessive pressure may increase people’s self-demands, resulting in higher perfectionism scores. However, some studies have not found a correlation between age and perfectionism (19), perhaps because this study relied on teenagers whose perfectionism traits may differ from those of adults [i.e., perfectionism seems to decrease after puberty (20)].

The study also found that high perfectionism predicted low self-esteem and high smile appearance concern, confirming Venet et al.’s findings that perfectionism negatively correlated with self-esteem but self-esteem is positively correlated with dental satisfaction (21). This may be because people with high perfectionism have higher standards for themselves, resulting in lower self-esteem scores and more smile appearance concerns. Excessive self-focus can lead to a mismatch between the expected self and the actual self, which may cause patients to evaluate themselves negatively, leading to lower self-esteem. Recent studies have shown that some specific cognitive processes partially mediate the relationship between perfectionism and self-esteem (22). Specifically, those with high perfectionism tend to use poor coping mechanisms (such as rumination), rather than active problem solving, which are unhelpful and may cause psychological distress.

In addition, this study found that high perfectionism predicted higher body image. Body image partly reflects the evaluation of orofacial appearance. A person who appreciates his/her own body is more likely to cooperate and listen to the doctor’s advice (8). For example, in an all-female sample, Zhang et al. found that body image appreciation may play a mediating role in the relationship between healthy perfectionism and body-related shame. Specifically, the healthier the perfectionism was, the more body image appreciation was felt, which reduced body-related shame (23). Consequently, we hope our finding will help dentists quickly gain a general understanding of patients’ self-perception.

This study also found that high perfectionism predicted high PHQ-4 scores, suggesting that college students with high perfectionism are more likely to suffer from anxiety and depression. A cohort study found that two dimensions of perfectionism, self-criticism and personal standards, moderated the negative effects of chronic stress on depression in 1 year (24). Partly consistent with the present study, people with high perfectionism showed higher scores of SOP and PHQ-4, as self-criticism and personal standards are two crucial parts of SOP. High self-criticism might be a negative self-cognition not leading directly to depression, but low self-esteem does seem to predict the development of depression (25). It could be suggested that high SOP tendency predicts low self-esteem, corroborating the present results.

With regard to dental treatment, only those who did not receive dental treatment showed no significant correlations in smile appearance concern and PHQ-4 stratifications. This may be caused by the two groups’ different expectations for their appearance. Studies have found a significant correlation between patients’ perfectionism and dental anxiety. Before aesthetic repair of anterior teeth or orthodontic treatment, patients showed high levels of depression and anxiety (26, 27). It is probable that people who actively participate in dental treatment tend to be dentally anxious and would pay more attention to their appearance and various symptoms, while people who are reluctant to undergo dental treatment may be as concerned about their appearance.

Pathway analysis indicated mental health as a significant mediator in the relationships between perfectionism and body image, smile appearance concern and self-esteem. It was revealed that more anxiety and depression could decrease the importance participants attached to their body image. Presumably, under conditions of extreme anxiety and depression, college students may have difficulty concentrating and become preoccupied with problems. Previous research has demonstrated that when faced with anxiety and overwhelming stress, college students may resort to behaviors such as praying, overeating, and seeking social support to cope, thereby neglecting their excessive attention to their oral appearance (28). The study also found that poorer mental health could lead to more sever smile appearance concern. Consistent with Emanuela et al. ‘s finding that depression could predict low body image satisfaction, college students with more anxiety and depression might tend to be dissatisfied with smile appearance (29). In addition, mental health was found to lower self-esteem. It was speculated that perfectionists tend to view life more negatively when experiencing more anxiety or depression, which exacerbates their negative self-evaluation (30).

There are several limitations to this study. First, our research was cross-sectional so may just provide a snapshot view of the constructs measured as opposed to long-lasting relationships. The PHQ-4 scale consists of only four questions, so longer, more sophisticated scales may provide a more nuanced view of mental illness. Every participant’s criteria for self-evaluation are inevitably different, so it is possible to set a standard on the first page of the questionnaire. Lastly, more factors could be included in the structural model of the mediation analysis for further investigation.

## 5. Conclusion

College students with high perfectionism are more likely to suffer from anxiety and depression and low self-esteem. Among college students who have received dental treatment, high perfectionism also predicted high body image, which may be useful for dentists to know so as to quickly understand patients’ perception of themselves. No significant correlation between perfectionism and smile appearance concern was found after adjusting for confounds. Mental health acted as a mediator in the relationships between perfectionism and body image, smile appearance concern, and self-esteem.

## Data availability statement

The raw data supporting the conclusions of this article will be made available by the authors, without undue reservation.

## Ethics statement

The studies involving human participants were reviewed and approved by Ethic approval for this research was obtained from West China School of Stomatology Sichuan University Medical Ethics Committee (Code: WCHSIRB-CT-2022-241). The patients/participants provided their written informed consent to participate in this study.

## Author contributions

XG and JZ: conceptualization, formal analysis, investigation, methodology, writing—original draft, and writing—review and editing. HL and YP: investigation, writing—review and editing. XL and SZ: investigation. YY: conceptualization, project administration, supervision, and funding acquisition. XX: conceptualization, project administration, resources, and supervision. All authors contributed to the article and approved the submitted version.

## Funding

This study was supported by Chengdu Science and Technology Bureau (no. 2022-YF05-01691-SN) and Clinical Research Project of West China Hospital of Stomatology, Sichuan University (no. LCYJ-2023-YY-2).

## Conflict of interest

The authors declare that the research was conducted in the absence of any commercial or financial relationships that could be construed as a potential conflict of interest.

## Publisher’s note

All claims expressed in this article are solely those of the authors and do not necessarily represent those of their affiliated organizations, or those of the publisher, the editors and the reviewers. Any product that may be evaluated in this article, or claim that may be made by its manufacturer, is not guaranteed or endorsed by the publisher.

## Footnotes

^1^ http://www.wjx.com
